# Oral health KAP and their association with OHRQoL among type 2 diabetic patients in the West Bank, Palestine: a cross-sectional study

**DOI:** 10.3389/froh.2025.1670923

**Published:** 2025-10-31

**Authors:** Iman Wahbeh, Aesha L. E. Enairat, Ihab Hemieid, Mahmoud Amro, Malak Abueed, Yazan Najem Hirzallah, Elham Kateeb

**Affiliations:** 1 Faculty of Graduate Studies, AL-Quds University, Jerusalem, Palestine; 2 Faculty of Medicine, Al-Quds University, Jerusalem, Palestine; 3 Faculty of Medicine and Health Sciences, An-Najah National University, Nablus, Palestine; 4 Oral Health Research and Promotion Unit, Al-Quds University, Jerusalem, Palestine

**Keywords:** diabetes, oral health-related quality of life, knowledge, attitude, practice, oral hygiene, OHIP-14

## Abstract

**Objectives:**

This study aimed to assess the impact of knowledge, attitudes, and practices (KAP) related to oral health on oral health-related quality of life (OHRQoL) among patients with type 2 diabetes mellitus (T2DM).

**Methods:**

A cross-sectional study was conducted from July 2023 to July 2024 in primary healthcare centers in the West Bank, using cluster sampling to select participants from three geographic regions A convenience sample was drawn from participants aged 40 years and older who were diagnosed with (T2DM). A structured validated Arabic questionnaire was employed to collect data on socio-demographic characteristics, oral health knowledge, attitudes, practices, and OHRQoL, using validated scales such as the OHIP-14.

**Results:**

The results showed that the mean OHRQoL score was 17.84 ± 11.65 (range 0–50), the primary domains negatively impacting participants' oral health-related quality of life were psychological discomfort, social disability, and handicap. Key oral health problems reported included dry mouth (62.2%), tooth loss (48.6%), and caries (46.1%). Knowledge scores averaged 6.53 ± 2.07 (range 1–10) attitudes scores were 4.88 ± 1.65 (range 0–6), and practices scores were 1.99 ± 1.02 (range 0–6). Spearman's correlation analysis revealed significant positive correlations between practice and knowledge (*ρ* = 0.160, *P* = 0.000), practice and attitude (*ρ* = 0.171, *P* = 0.000), and Knowledge and attitude (*ρ* = 0.238, *P* = 0.000). In the final model, predicting factors to improve OHRQoL were full-time employment, better income, and positive attitude, while poorer OHRQoL was predicted by pain reason to visit dentist, discussion with a dentist about diabetes and oral complications, poor general health status, poor oral health status, lower educational level, no history of diabetes and long duration of to do HbA1c test.

**Conclusion:**

This study highlights that positive attitudes significantly improve OHRQoL in diabetic patients, while poor outcomes relate to socioeconomic and health system barriers. Despite good knowledge, practices remain inadequate. Integrating oral health into diabetes care, improving access, and addressing social determinants are essential for enhancing overall quality of life in this population.

## Introduction

Oral health is closely connected to overall health, as both share common risk factors such as tobacco use, poor diet, physical inactivity, and alcohol consumption (1, 2). Oral diseases are strongly linked to the four major non-communicable diseases (NCDs)—cardiovascular disease, cancer, diabetes, and chronic respiratory illnesses (1, 2). Recognizing this, the World Health Organization (WHO), in its 2022 Global Oral Health Status Report, emphasized the critical need to integrate oral health into strategies addressing NCDs and Universal Healthcare Coverage (UHC) (3). This integration is vital to ensuring comprehensive healthcare that addresses broader determinants of health, improves outcomes for individuals with NCDs, and reduces the global burden of oral diseases.

Diabetes Mellitus (DM) is a chronic metabolic condition that develops when the pancreas cannot produce insulin or when the body cannot effectively use the insulin produced (4). Insulin regulates blood glucose levels (4), and the aetiology of DM is multifactorial, involving both genetic and environmental factors (5). The American Diabetes Association (ADA) classifies diabetes into different types, including type 1 diabetes (insulin deficiency) and type 2 diabetes (insulin resistance) (6). In 2021, the International Diabetes Federation (IDF) reported that 537 million people aged 20–79 had diabetes worldwide, a number projected to increase to 643 million by 2030, 783 million by 2045 (7), and 1.31 billion by 2050 (8). Type 2 diabetes mellitus (T2DM) accounts for 90%–95% of all cases globally (9).

For most adults with diabetes, uncontrolled disease is indicated by an HbA1c of 7% or higher, while values above 9% represent very poor control (10). Poorly managed diabetes can result in severe complications such as kidney disease, cardiovascular disease, and diabetic foot ulcers (11). Oral health complications are also common, including gingivitis, periodontitis, xerostomia, fungal infections, plaque accumulation, delayed wound healing, and altered taste (11). These issues are largely associated with diabetes-related microvascular and macrovascular damage (11). Hyperglycaemia has been shown to share a bidirectional relationship with periodontal disease: poor glycaemic control worsens periodontal status, while periodontal inflammation can impair diabetes management (12). Preventive measures—such as regular toothbrushing, flossing, dental visits, and smoking cessation—can mitigate these complications (9, 13). Nevertheless, adherence to such practices is often hindered by limited knowledge, financial barriers, and restricted access to dental care, particularly in underserved populations (14).

Health-related quality of life (HRQoL) reflects how illness and its management affect patients' physical, psychological, and social well-being (15). Within this framework, oral health-related quality of life (OHRQoL) has emerged as an important yet underexplored dimension, highlighting the impact of oral conditions on daily life (16). Most studies suggest a negative association between diabetes and OHRQoL (17, 18), though some report no significant effect (19, 20). Effective dental management and patient education remain essential for reducing oral complications and improving OHRQoL.

Patients' knowledge, attitudes, and practices (KAP) related to oral health are key determinants of OHRQoL, offering valuable insights for preventive strategies and awareness programs (21). A scoping review of diabetes in South Asia found that strengthening knowledge and fostering positive attitudes can enhance oral health practices (22). Moreover, lower education levels and poorer general health are linked to reduced OHRQoL among the elderly (23), while higher education correlates with better health knowledge in diabetic populations (24). Despite this, evidence shows that many individuals with diabetes have limited awareness of oral complications and receive inadequate oral health guidance from their care providers (22). Knowledge of periodontal risk is often lower than knowledge of other complications (25), and patients are generally more informed about systemic complications than oral manifestations (12).

Although studies in Palestine and neighbouring regions have examined diabetes-related KAP, general quality of life (26–28), and oral health in diabetic patients (29, 30), these aspects have largely been studied in isolation. No research in Palestine has specifically assessed how oral health-related KAP influences OHRQoL among patients with T2DM. Addressing this gap is critical, as better understanding these associations can guide targeted health education, improve preventive strategies, and inform Ministry of Health (MoH) policies.

Therefore, the present study aims to evaluate the relationship between oral health-related KAP, oral health behaviours, and OHRQoL among T2DM patients attending MoH primary healthcare centers in the West Bank. Additionally, it seeks to explore the influence of demographic, socioeconomic, and dental care access factors on these outcomes, and to analyse different domains of the OHIP-14 in relation to overall OHRQoL.

## Methods

### Study design and setting

A cross-sectional study was conducted in the primary healthcare centers (PHCs) of the Palestinian Ministry of Health (MoH) in the West Bank from July 2023 to July 2024. The West Bank was selected due to logistical feasibility, the concentration of MoH PHCs, and the availability of reliable patient records, which facilitated standardized data collection. While this limits generalizability to other Palestinian regions such as Gaza, findings are expected to be broadly indicative of the West Bank population.

### Sampling strategy

**Figure 1:**
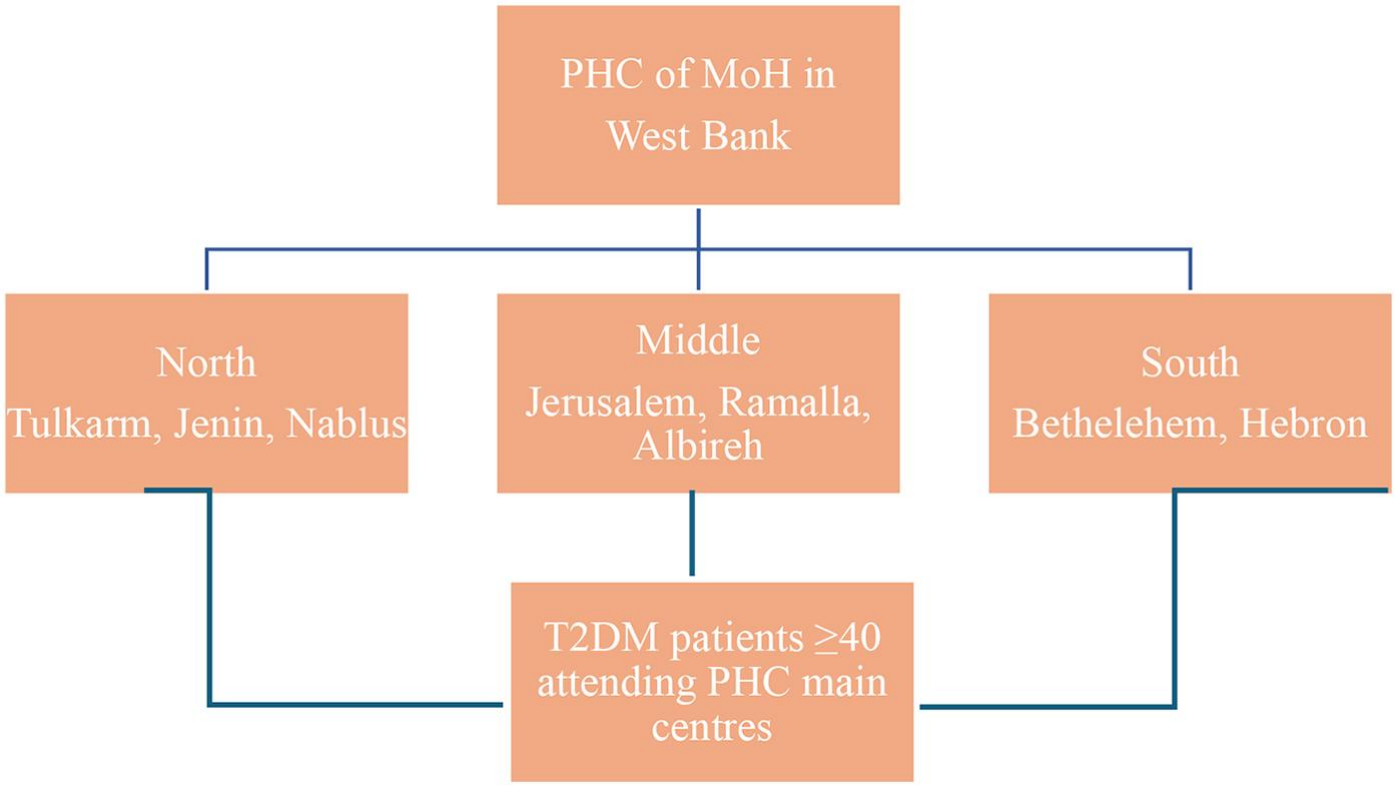
Flowchart of sample technique.

A cluster sampling approach was used to select governorates representing the three main geographic areas of the West Bank: North, Central, and South. The selected governorates were Jenin, Nablus, and Tulkarm (North); Jerusalem, Ramallah, and Al-Bireh (Central); and Bethlehem and Hebron (South) (Figure 1). PHCs within each governorate were purposively selected based on patient volume, prioritizing clinics with the highest number of registered type 2 diabetes mellitus (T2DM) patients to ensure adequate recruitment.

The West Bank has approximately 493 in total primary healthcare centers (PHCs), accounting for 64.3% of all healthcare centers in the country (31), with 375 PHCs in the selected governorates;10 centers were purposively selected based on the highest patient volume of type 2 diabetes mellitus (T2DM) patients, to ensure efficient recruitment and adequate sample size. The selected centers included:

- North: Jenin main PHC, Nablus main PHC and another PHC clinic, Tulkarm main PHC.
- Central: Ramallah (Al-Bireh) main PHC, Jerusalem main PHC.
- South: Bethlehem main PHC, three main PHC clinics in Hebron.

Participants were recruited consecutively from these centers. Inclusion criteria were patients aged 40 years or older, diagnosed with T2DM for at least 6 months, and able to provide informed consent. Exclusion criteria were patients with type 1 diabetes mellitus (T1DM), prediabetes, gestational diabetes, secondary diabetes, or undiagnosed diabetes were excluded.

### Sample size calculation

Based on an estimated T2DM prevalence of 20% in the West Bank [Markov model study (32)] and a population of 3.25 million (Palestinian Central Bureau of Statistics, 2023), the minimum required sample size was calculated using the Epitools online calculator with a 5% margin of error and 95% confidence level. The estimated sample size was 457, which was increased to 508 to account for a potential 10% nonresponse rate.

### Conceptual framework

**Figure 2:**
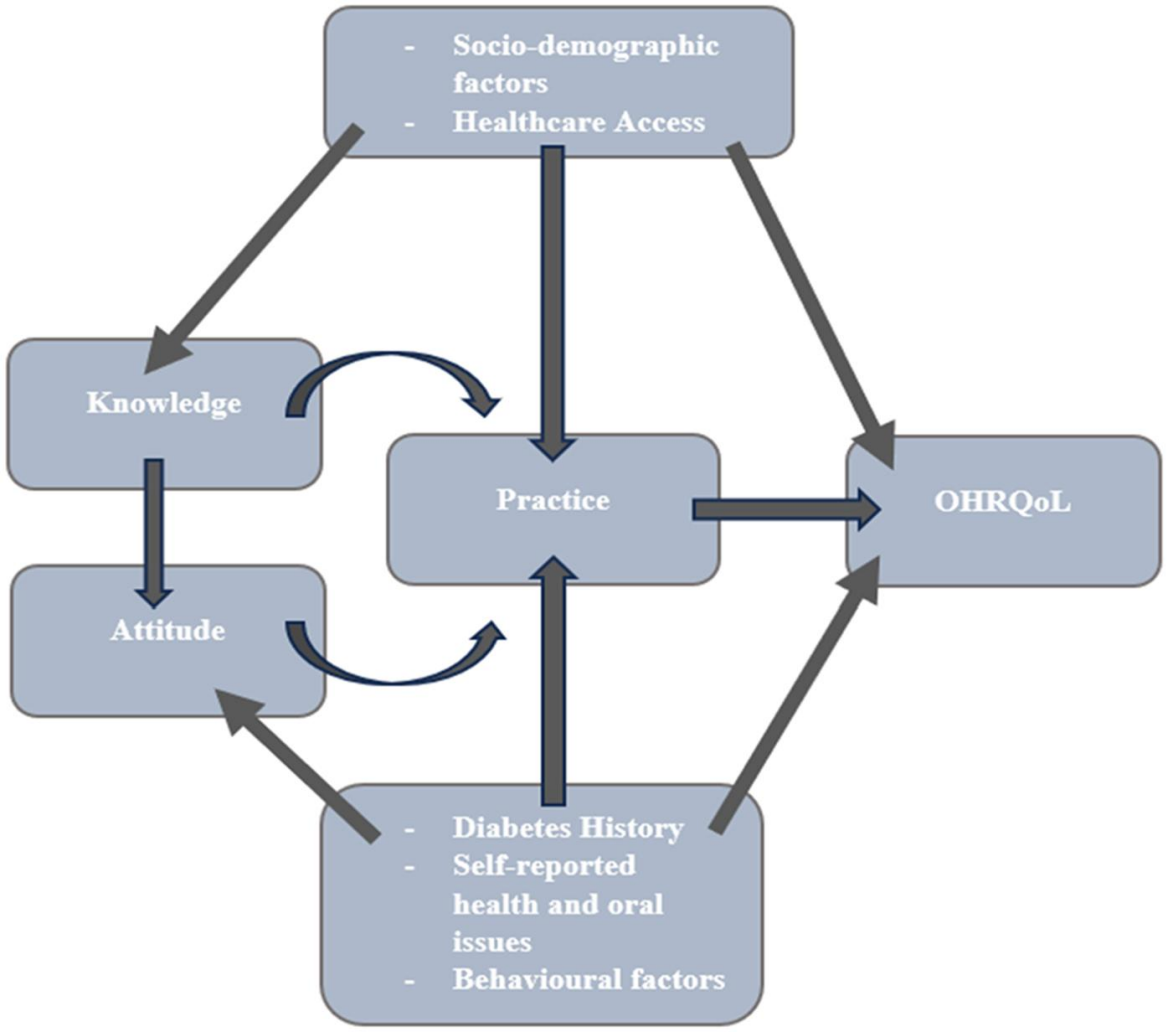
Conceptual framework for study variables.

The study's conceptual framework (Figure 2) was adapted from previously published models on oral health-related quality of life (OHRQoL), diabetes knowledge, attitudes, and practices (KAP) (33–35). It was not newly developed but contextualized to the Palestinian setting, with cultural and demographic adjustments. All adaptations were based on validated instruments referenced in the literature.

### Data collection and study tool

Data were collected through in-person structured interviews using a pre-tested Arabic questionnaire implemented via Google Forms. Participants were briefed on study aims, benefits, and confidentiality, and verbal informed consent was obtained. The study was approved by the Al-Quds University Ethics Committee and the Palestinian MoH.

The questionnaire had five sections covering demographics, diabetes history, KAP, dietary habits, oral hygiene practices, complications, and OHRQoL. The OHIP-14 instrument, previously validated in Arabic (36, 37), measured OHRQoL. Forward-backward translation ensured cultural and linguistic accuracy, and expert review (two dentists, a nurse researcher, and a public health researcher) refined the questions. Pilot testing with 45 patients demonstrated readability, comprehension, and internal consistency (Cronbach's *α* = 0.87).

### HbA1c measurement

HbA1c data were obtained from patient medical records at each PHC, reflecting the most recent laboratory measurement within the past 3 months. This ensured standardized and accurate glycaemic control data for all participants.

### Data collector training and qualifications

Field researchers were selected based on prior experience in healthcare data collection, educational background in medical, research and public health, and proximity to target governorates. All were trained in standardized interview techniques through two Zoom calibration sessions led by the principal investigator. Ongoing supervision was provided via phone and messaging to ensure consistency in data collection, ethical conduct, and adherence to study protocols.

### Data analysis

#### Descriptive & normality testing

Means, SDs, frequencies, and percentages summarized participants' demographics, diabetes history, health status, and healthcare access. Normality of KAP (Knowledge, Attitude, Practice) and OHRQoL was tested using Kolmogorov–Smirnov and Shapiro–Wilk; *p* < 0.05 indicated non-normality, so non-parametric tests were applied. Analyses were conducted in IBM SPSS 26.0.

#### Scoring & composite variables

Knowledge: Correct = 1, Incorrect = 0. Total 0–10 (Higher = better knowledge). Attitude: “Agree/Totally agree” = 1; others = 0. Total 0–6 (Higher = positive attitude). Oral Hygiene Practices: Toothbrushing/flossing ≥1/day, correct technique, 2 min brushing, and fluoride/mouthwash use = 1; others = 0. Total 0–6 (Higher = better hygiene). Dietary Habits: Recoded to reflect poorer habits with higher scores (0 = good, 1 = fair, 2 = poor). Oral & General Complications: Yes = 1, No/Not sure = 0. (Higher = more complications). OHRQoL (OHIP-14): Score 0–56 (Higher = poorer quality of life), for the 7 domains, each 0–8 scores (Higher = poorer quality of life).

#### Bivariate analysis

Spearman's correlation, Mann–Whitney *U* and Kruskal–Wallis tests were used.

#### Multivariate analysis

Stepwise multiple linear regression assessed predictors of OHRQoL (continuous). Predictors: significant bivariate variables + theoretically relevant ones (Knowledge, Practice, Age, Smoking, Family history, Last dental visit, Education program attendance). Categorical predictors were dummy-coded; 51 predictors included. Stepwise results confirmed with forward regression.

#### Collinearity & model fit

No multicollinearity (VIF < 1.21, Tolerance > 0.82). Model fit evaluated via adjusted R^2^. Significance set at *p* < 0.05 (two-tailed).

## Results

### Socio-demographic and behavioural characteristics of participants

**Figure 3:**
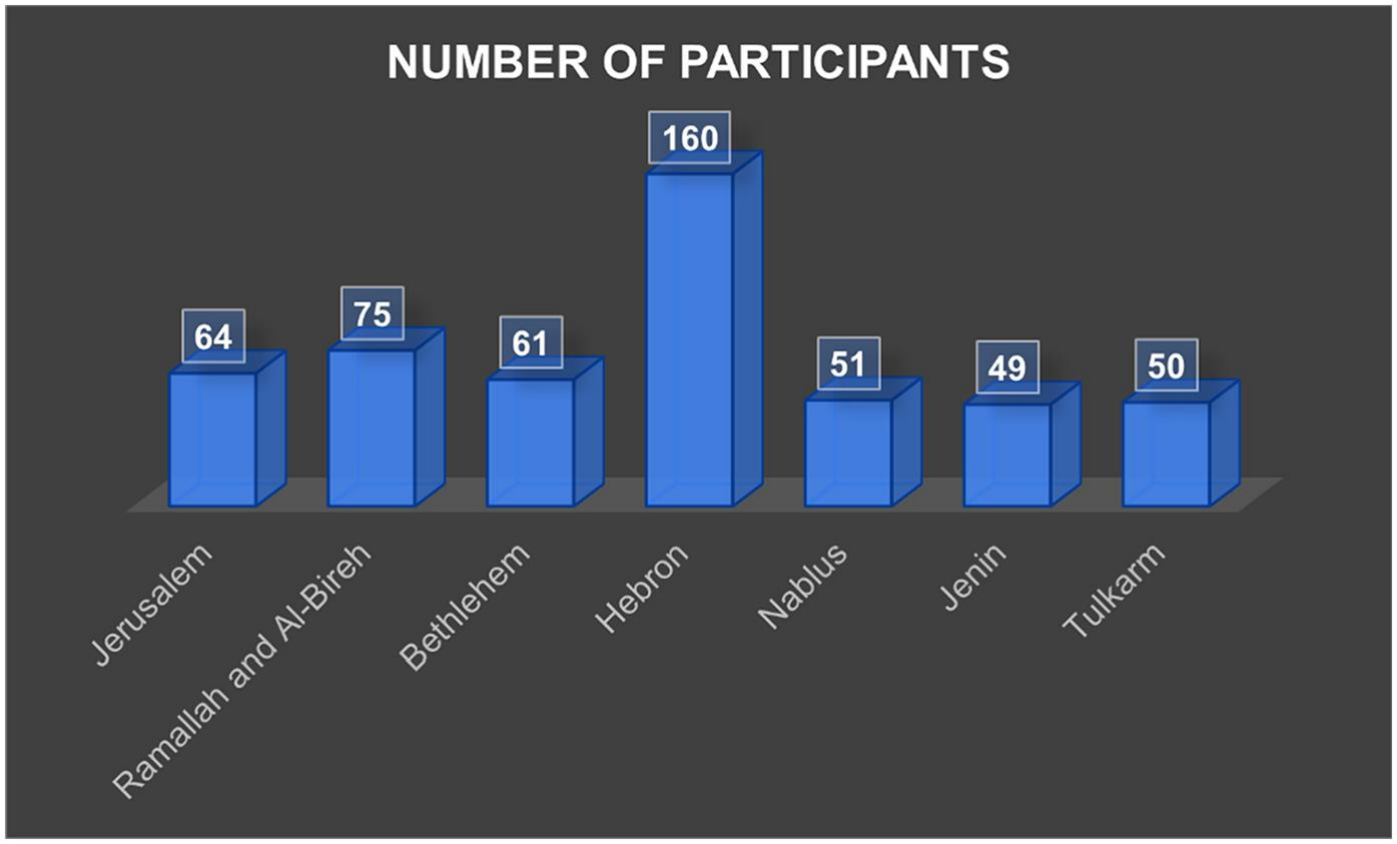
Number of participants in each governorate.

A total of 510 participants completed the study. The mean age was 60.4 ± 9.3 years (range 40–85), with 58.2% females. Hebron contributed the largest proportion of participants (31.4%), consistent with its size and distribution of PHCs (Figure 3). Most participants were married (75.5%), lived in cities (52.7%), and had completed high school or higher education (46.5%). Monthly household income was below $500 for half of the sample (50.6%). Current smokers comprised 26.1%, and 40.8% reported regular consumption of fruits and vegetables, whereas 49.2% reported frequent sugar intake (Tables 1, 2).

**Table 1:** Sociodemographic characteristics of the participants.

Sociodemographic characteristics	Categories	Number of participants (*N*)	Percentage (%)
Gender	Male	213	41.8
	Female	297	58.2
Marital status	Married	385	75.5
	Divorced	18	3.5
	Widowed	86	16.9
	Single	21	4.1
Place of residence	Village	212	41.6
	City	269	52.7
	Camp	29	5.7
Educational level	Uneducated	37	7.3
	Elementary (1–6)	88	17.3
	Secondary (7–10)	100	19.6
	High School (11–12)	131	25.7
	2-years College	48	9.4
	4-years University and above	106	20.8
Monthly household Income (USD $)	Less than 250	130	25.5
	250–499	128	25.1
	500–799	126	24.7
	800–1,000	65	12.7
	More than 1,000	61	12
Employment	Full-time employee	98	19.2
	Part-time employee	36	7.1
	Unemployed	114	22.4
	Retired	75	14.7
	Housewife (Stayed home mother)	187	36.7

*N*, frequency; %, percentage.

**Table 2:** Health related behaviours among study participants.

Health related behaviours	Categories	*N*	%
Smoking	Current smoker	133	26.1
	Former smoker	80	15.7
	Never smoker	297	58.2
Type of smoking	Pipe	3	0.6
	Cigarettes	114	22.4
	Hookah	37	7.3
	Electronic cigarettes	1	0.2
	More than one type	10	2
	Not applicable*	345	67.6
Duration of smoking	1–5 years	13	2.5
	6–10 years	27	5.3
	11–15 years	19	3.7
	More than 15 years	90	17.6
	Not applicable*	361	70.8
Thinking of Quitting smoking	Yes	61	12
	No	83	16.3
	Not applicable*	366	71.8
Duration of quitting for a former smoker	1–5 years	38	7.5
	6–10 years	20	3.9
	11–15 years	9	1.8
	More than 15 years	14	2.7
	Not applicable*	429	84.1
Reason for quitting for a former smoker	Health reason	52	10.2
	Relatives ‘advice	4	0.8
	Family reason	12	2.4
	Economic reason	9	1.8
	Other (Will, Self-conviction)	2	0.4
	Not applicable*	431	84.5
Dietary Habits			
Eating vegetables and fruits	Good	208	40.8
	Fair	174	34.1
	Bad	128	25.1
Eating sugar and sweets	Good	91	17.8
	Fair	168	32.9
	Bad	251	49.2

*N*, frequency; %, percentage.

Not applicable* means a question or variable does not apply to a specific participant or situation.

### Patient's diabetes history, self-reported health issues and self-reported oral complications

A majority (70.2%) had a first-degree relative with diabetes. Mean HbA1C was 8.12 ± 1.78, with 64.5% of participants classified as uncontrolled. Most participants were on diabetes medication (92.9%), primarily oral hypoglycaemics. Common comorbidities included hypertension (65.9%) and hypercholesterolemia (47.6%). Oral complications were frequent, with xerostomia (62.2%), tooth loss (48.6%), and caries (46.1%) being most reported (Tables 3, 4).

**Table 3:** Self-reported health issues.

Self-reported health issues or chronic disease	Yes *N* (%)
High blood pressure	336 (65.9)
Cholesterol	243 (47.6)
Eyes problem-related diabetes	227 (44.5)
Arthritis (Rheumatism)	176 (34.5)
Cardiovascular disease	165 (32.4)
Respirator disease (Asthma, else)	74 (14.5)
Other endocrine disease	56 (11)
Stroke	45 (8.8)

*N*, frequency; %, percentage.

**Table 4:** Self-reported oral and dental complications.

Self-reported oral and dental issues	Yes *N* (%)	No *N* (%)	Not sure *N* (%)
Dry mouth (Xerostomia)	317 (62.2)	173 (33.9)	20 (3.9)
Loss of teeth	248 (48.6)	259 (50.8)	3 (0.6)
Caries (Tooth decay)	235 (46.1)	247 (48.4)	28 (5.5)
Bad breath (Halitosis)	178 (34.9)	303 (59.4)	29 (5.7)
Gingival bleeding	156 (30.6)	327 (64.1)	27 (5.3)
Change in taste	149 (29.2)	332 (65.1)	29 (5.7)
Stomatitis (infection or inflammation)	103 (20.2)	375 (73.5)	32 (6.3)
Candidiasis (Fungal infection)	90 (17.6)	393 (77.1)	27 (5.3)
Ulcers, abscesses, and teeth sensitivity	73 (14.3)	391 (76.7)	46 (9)
Burning mouth syndrome	71 (13.9)	405 (79.4)	34 (6.7)

*N*, frequency; %, percentage.

### Access to dental care and to information about oral health

Nearly all participants had insurance (95.9%), predominantly public. Satisfaction with PHC services was moderate (72.2%), with medication availability and waiting times as main concerns. Only 17.6% attended diabetes education programs, and 43.5% discussed diabetes with their dentist (Table 5).

**Table 5:** Access to healthcare service, satisfaction with provided services and access to health and oral health information.

Healthcare access variables	Categories	*N*	(%)
Health Insurance	Yes	489	95.9
	No	9	1.7
	Don't know	12	2.4
Type of Insurance	Public (Governmental)	440	86.3
	Private	38	7.5
	Don't know	26	5.1
	Other	6	1.2
Last dental visit	Less than 6 months	123	24.1
	6–12 months	92	18
	More than one year	198	38.8
	I don't visit the dentist	97	19
Reason for last dental visit	Regular checkup	55	10.8
	Cosmetic treatments	74	14.5
	Therapeutic visit	75	14.7
	Pain relief	139	27.3
	Other treatments	68	13.3
	I don't visit the dentist	99	19.4
Satisfaction with healthcare services in PHC	Satisfied	368	72.2
	Not satisfied	89	17.5
	Refused to answer	53	10.4
Attended educational programs regarding diabetes	Yes	90	17.6
	No	420	82.4
Talked to your physician about diabetes	Yes	164	32.2
	No	346	67.8
Talked to your dentist about diabetes	Yes	222	43.5
	No	288	56.5

*N*, frequency; %, percentage.

### Knowledge, attitude, and hygiene practice (KAP) related to diabetes

Mean composite scores were knowledge 6.53 ± 2.07, attitude 4.88 ± 1.65, and oral hygiene practices 1.99 ± 1.02. Higher knowledge and positive attitudes were significantly correlated with better oral hygiene practices (*ρ* = 0.160–0.238, *p* < 0.001). Education, income, city residence, regular dental visits, and attending educational programs were the strongest predictors of better KAP (Tables 6–8).

**Table 6:** Oral health knowledge questions and their correct answers proportions.

Question	Answer correctly *N* (%)	Answer incorrectly *N* (%)
People with diabetes are more susceptible to gum disease and dental-supporting tissues	372 (72.9)	138 (27.1)
There is no relationship between gum disease and dental supporting tissues and increased blood sugar levels in the blood	318 (62.4)	192 (37.6)
Diabetes does not cause bad breath	292 (57.3)	218 (42.7)
People with diabetes are more likely to have dry mouth	409 (80.2)	101 (19.8)
Diabetics usually do not suffer from oral thrush	293 (57.5)	217 (42.5)
Diabetics have a higher incidence of tooth decay due to dry mouth	323 (63.3)	187 (36.7)
Diabetes is not a cause of tooth loss in patients	318 (62.4)	192 (37.6)
Regulating blood sugar levels may protect against oral and dental diseases in diabetics	374 (73.3)	136 (26.7)
Smoking is not a risk factor for increased oral and dental diseases in diabetics	359 (70.4)	151 (29.6)
There is a relationship between chronic gum inflammation and cardiovascular disease in diabetics	272 (53.3)	238 (46.7)

*N*, frequency; %, percentage.

**Table 7:** Oral health related attitude statements and their ratings.

Statement	Totally agree *N* (%)	Agree *N* (%)	Neutral *N* (%)	Disagree *N* (%)	Totally disagree *N* (%)
Taking care of your mouth is as important as taking care of other parts of your body	305 (59.8)	155 (30.4)	31 (6.1)	15 (2.9)	4 (0.8)
It is important to brush your teeth in the morning and before going to bed	257 (50.4)	166 (32.5)	58 (11.4)	23 (4.5)	6 (1.2)
I think it is necessary to maintain a visit to the dentist at least once a year for a regular check-up of the mouth and teeth	224 (43.9)	145 (28.4)	84 (16.5)	46 (9)	11 (2.2)
If you have a problem with your mouth or teeth, you should consult a dentist	255 (50)	152 (29.8)	66 (12.9)	30 (5.9)	7 (1.4)
It is important for the medical team supervising diabetic patients to provide information about the complications of diabetes on oral and dental health during a routine visit to the diabetes clinic	275 (53.9)	160 (31.4)	62 (12.2)	8 (1.6)	5 (1)
It is important that your doctor who treats your diabetes to refer you to a dentist for a regular oral and dental check-up	237 (46.5)	158 (31)	64 (12.5)	26 (5.1)	25 (4.9)

*N*, frequency; %, percentage.

**Table 8:** Oral hygiene practices frequencies.

Practice	At least once a day	Sometimes	Rarely	Never
Do you brush your teeth?	275 (53.9)	117 (22.9)	60 (11.8)	58 (11.4)
Do you floss?	26 (5.1)	54 (10.6)	45 (8.8)	385 (75.5)
**Practice**	**Yes**	**Sometimes**	**I don't know**	**No**
Do you use fluoride toothpaste to clean your teeth?	94 (18.4)	25 (4.9)	282 (55.3)	109 (21.4)
Do you use mouthwash?	7 (1.4)	78 (15.3)	97 (19)	328 (64.3)
**Practice**	**Two minutes**	**Less than 2 min**	**More than 2 min**	**I don't know/I don't brush my teeth**
How long do you spend brushing your teeth?	98 (19.2)	220 (43.1)	65 (12.7)	127 (24.9)
**Practice**	**Correct**		**Incorrect**	
How do you hold your toothbrush when brushing your teeth, can you show me? (The correct way is at a 45-degree angle so that the bristles of the brush touch the tip of the gums and teeth)	176 (34.5)		334(65.5)	

*N*, frequency; %, percentage.

### Oral health-related quality of life (OHRQoL)

**Figure 4:**
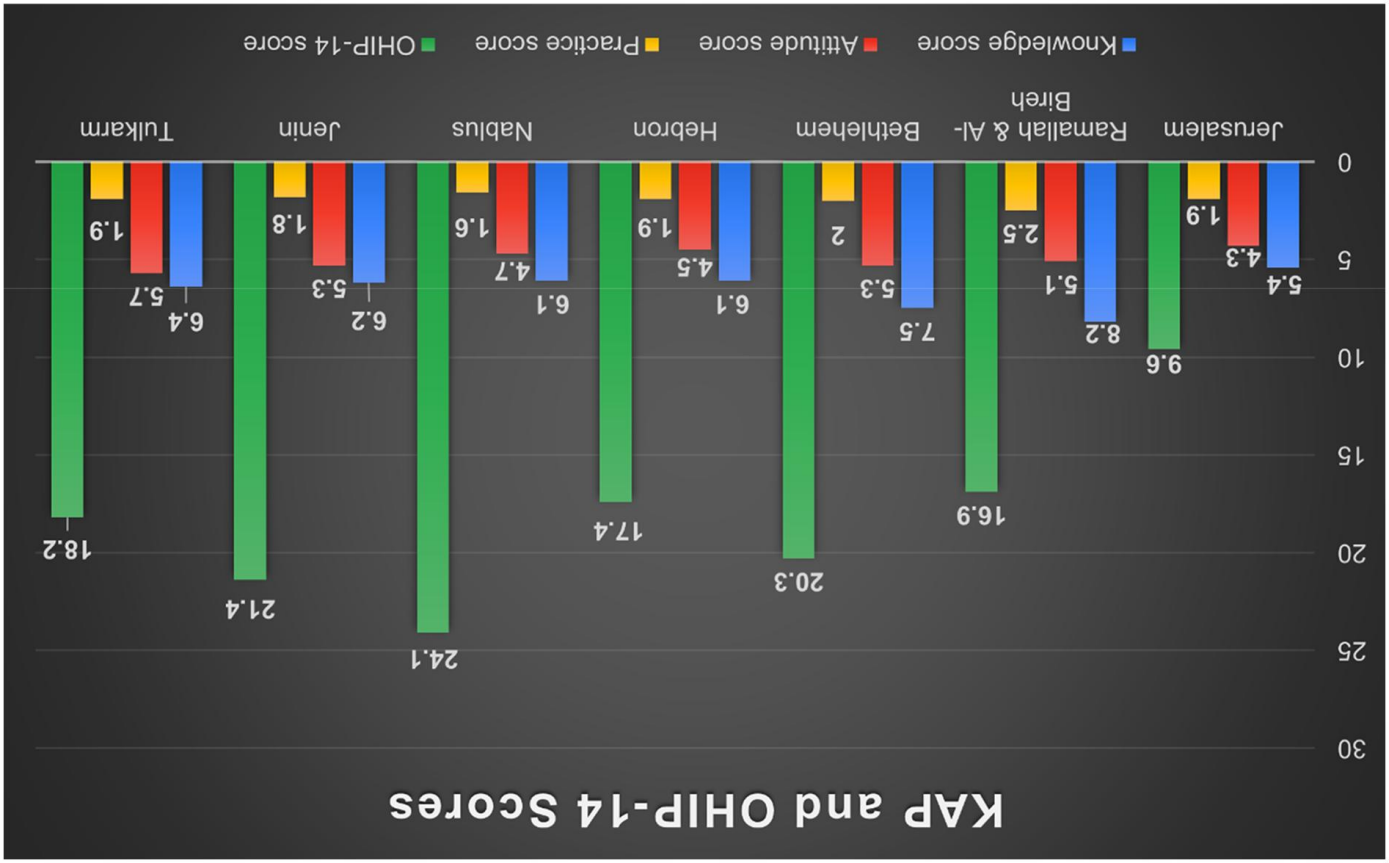
KAP and OHIP-14 scores distribution according to governorates.

Mean OHIP-14 score was 17.84 ± 11.65, indicating moderate impact. Psychological discomfort, social disability, and handicap domains were most affected (Table 9). While (Figure 4) illustrates the distribution of KAP and OHIP-14 scores across governorates.

**Table 9:** OHIP-14 scale entries that negatively impacted participants (*N* = 510).

Domain	Questions	*N* (%)
Functional limitation	Trouble pronouncing words	9 (1.8)
	Worsened taste	16 (3.1)
Physical pain	Pain in mouth	18 (3.5)
	Discomfort eating food	24 (4.7)
Psychological discomfort	Feeling self-conscious	86 (16.9)
	Feeling tense	72 (14.1)
Physical disability	Interrupted meals	29 (5.7)
	Poor diet	18 (3.5)
Psychological disability	Difficulty relaxing	65 (12.7)
	Embarrassment	53 (10.4)
Social disability	Irritability	82 (16.1)
	Difficulty in doing usual jobs	79 (15.5)
Handicap	Life less satisfying	82 (16.1)
	Inability to function	80 (15.7)

*N*, frequency; %, percentage.

### Bivariate analysis

Spearman's correlation revealed significant positive relationships between Knowledge, Attitudes, and Practices:

Knowledge ↔ Oral hygiene practices (*ρ* = 0.160, *p* < 0.001)
Attitudes ↔ Oral hygiene practices (*ρ* = 0.171, *p* < 0.001)
Knowledge ↔ Attitudes (*ρ* = 0.238, *p* < 0.001)

These findings suggest that better knowledge and positive attitudes are linked to improved oral health practices.

### Predictors of better oral health knowledge

Clinically relevant predictors of higher knowledge included:

Younger age (*ρ* = −0.190, *p* < 0.001)
Urban residence [H(2) = 11.4, *p* < 0.001]
Higher education (*ρ* = 0.263, *p* < 0.001) and higher income (*ρ* = 0.121, *p* < 0.05)
Full-time employment [H(4) = 12.1, *p* < 0.05]
Regular dental visits, especially routine check-ups (H = 38.6–56.2, *p* < 0.001)
Attending diabetes education programs (*U* = 23,034.5, *p* < 0.001)
Discussing oral health with healthcare providers (physician: *U* = 33,341.5, dentist: *U* = 39,829, *p* < 0.001)

Lower knowledge was associated with longer diabetes duration and extended intervals since the last dental visit.

### Predictors of positive attitudes

Positive attitudes toward oral health were more likely among:

Younger participants (*ρ* = −0.142, *p* < 0.001) and females (*U* = 35,820.5, *p* < 0.05)
Urban residents [H(2) = 20.9, *p* < 0.001]
Higher education and income (*ρ* = 0.189, *H* = 21.1–23.7, *p* < 0.001)
Full-time employment [H(4) = 12.4, *p* < 0.05]
Healthy diet and non-smoking (*H* = 9.48–28.3, *p* < 0.001)
Controlled diabetes and family history of diabetes [*U* = 25,484.5; H(3) = 13.59, *p* < 0.05]
Regular dental visits and satisfaction with healthcare services (*H* = 7.06–48.7, *p* < 0.05)
Engagement in educational programs and discussions with providers (*U* = 22,485.5–38,038, *p* < 0.001)

Negative predictors included older age, longer diabetes duration, higher HbA1C, cumulative sugar results, and poorer general health.

### Predictors of favourable oral hygiene practices

Better oral hygiene practices were associated with:

Higher education and income (*ρ* = 0.188–0.282, *p* < 0.001)
Full-time employment [H(4) = 34.7, *p* < 0.001]
Younger age (*ρ* = −0.278, *p* < 0.001)
Regular dental visits, particularly for check-ups or therapeutic reasons (*H* = 26.4, *p* < 0.001)
Controlled diabetes (*U* = 25,922.5, *p* < 0.05)

Negative predictors included longer diabetes duration, poorer general health, higher cumulative sugar test results, and infrequent dental visits.

### Predictors of oral health-related quality of life (OHRQoL)

Poorer OHRQoL (higher OHIP-14 scores) was associated with:

Female gender (*U* = 36,287.5, *p* < 0.05)
Unemployment [H(4) = 25.35, *p* < 0.001]
Uncontrolled diabetes (higher HbA1C, *U* = 34,421.5, *p* < 0.05)
Worse general health and more diabetes-related oral health issues (*ρ* = 0.252–0.407, *p* < 0.001)
Dental visits for pain relief or discussions about diabetes with dentists/physicians (*U* = 32,919–36,925, *H* = 25.65, *p* < 0.05)

Better OHRQoL was observed in participants with higher education and income, positive attitudes, better oral hygiene practices, and controlled diabetes.

### OHIP-14 domains

Across the seven OHIP-14 domains, clinically relevant findings included:

Functional Limitation & Physical Pain: Worse outcomes in participants who had not attended educational programs or consulted healthcare providers, and those visiting dentists for pain relief or never visiting.
Psychological Discomfort & Disability: Associated with higher HbA1C, more oral health issues, and poorer general health.
Social Disability & Handicap: Worse outcomes in participants attending educational programs, discussing diabetes with providers, or visiting for pain relief, but better outcomes among those with higher education, income, and positive attitudes.

### Bivariate analysis of the different domains of OHIP-14

Significant differences (*p* < 0.05) in mean scores across the seven domains of OHIP-14 were identified using Mann–Whitney and Kruskal–Wallis tests. These differences were associated with participants' engagement in diabetes-related educational programs, discussions with healthcare providers about their condition, and the reason for their recent dental visit.

### OHIP-14 domain outcomes

Analysis of OHIP-14 domains revealed several consistent predictors of oral health-related quality of life (OHRQoL) among participants.

**Functional limitation** was significantly worse among individuals who had not attended diabetes education programs, had not discussed their condition with healthcare providers, or sought dental care only for pain. Elevated HbA1c, self-reported diabetes-related oral complications, and poorer general health were also linked to greater limitations, whereas positive attitudes toward oral health were protective.

**Physical pain** was more pronounced in participants with uncontrolled diabetes, oral complications, and poor health status, while higher education and preventive dental visits were associated with less pain.

**Psychological discomfort** was unexpectedly higher among those who attended education programs or discussed their condition with physicians or dentists, likely reflecting heightened awareness of oral health risks. Discomfort was further exacerbated by poor glycemic control, oral complications, and poor health status, but mitigated by higher income and more frequent dental visits.

**Physical disability** was strongly associated with poor glycemic control, oral complications, and lower socioeconomic status. Protective factors included higher income, positive attitudes, and better oral hygiene practices.

**Psychological disability** was more common among participants with higher HbA1c, poor general health, and oral complications, but was significantly reduced in those with higher education and income.

**Social disability** was elevated among participants with uncontrolled diabetes, oral complications, or lower socioeconomic status. By contrast, higher education and income were associated with reduced social disability.

**Handicap** scores were higher among older participants and those with poor glycemic control, poor health status, or oral complications. In contrast, higher education, better income, positive attitudes, and healthier oral practices were protective.

### Overall predictors of OHRQoL

Across domains, three predictors consistently emerged as the most clinically relevant for poorer OHRQoL:

- Poor glycemic control (higher HbA1c levels).
- Presence of diabetes-related oral complications.
- Lower socioeconomic status (education and income).

Conversely, positive attitudes, healthier oral practices, and proactive dental care were protective across multiple domains.

### Multivariable analysis for OHRQoL variable (OHIP-14 scores)

A total of 51 variables were entered into multiple linear regression with stepwise method and confirmed with forward method. Eleven models were produced, and model number 11 with eleven variables was selected with R^2^ was 0.306 and adjusted R^2^ was 0.290, *p* value < 0.000 and F change was 19.95. Predicted variables for poor or good oral health related quality of life are shown in Table 10.

**Table 10:** Multiple linear regression for the dependent variable OHRQoL (OHIP-14 scores) (*N* = 510).

Variables	Standardized *β*	*T*	*P*-value	95% confidence interval for *β*	
				Upper	Lower
Constant		5.802	0.000	7.468	15.115
Total Oral issues	0.299	7.453	0.000	1.114	1.911
Full-time employee	−0.125	−3.169	0.002	−6.000	−1.407
Talked to dentist	0.200	5.049	0.000	2.864	6.514
Elementary education	0.124	3.179	0.002	1.456	6.166
Pain relief visit	0.139	3.517	0.000	1.604	5.664
No DM in family	0.126	3.291	0.001	1.533	6.077
Total Attitude score	−0.103	−2.615	0.009	−1.279	−0.182
800$–1,000$ per month	−0.128	−3.269	0.001	−7.170	−1.787
HbA1C test >3 months	0.098	2.534	0.012	0.542	4.282
Total health issues	0.104	2.567	0.011	0.175	1.320
500$–800$ per month	−0.079	−1.978	0.048	−4.246	−0.014

OHRQoL-Oral Health Related Quality of Life-Dependent variable.

*β* Standardization beta coefficient, *t* statistics of standard error.

Stepwise regression model fit Adjusted R Squared was 0.290, *p* < 0.000.

### Factors associated with poorer OHRQoL (higher OHIP-14 scores)

- Presence of diabetes-related oral complications (*β* = 0.299, *p* < 0.001).
- Poorer general health status (*β* = 0.104, *p* < 0.05).
- Having discussed diabetes with a dentist (*β* = 0.200, *p* < 0.001).
- Lower educational level (elementary only) (*β* = 0.124, *p* < 0.001).
- Dental visits primarily for pain relief (*β* = 0.139, *p* < 0.001).
- Lack of family history of diabetes (*β* = 0.126, *p* < 0.001).
- Delayed HbA1c monitoring (>3 months) (*β* = 0.098, *p* < 0.05).

### Factors associated with better OHRQoL (lower OHIP-14 scores)

- Full-time employment (*β* = −0.125, *p* < 0.05).
- Positive attitudes toward oral health (*β* = −0.103, *p* < 0.05).
- Higher monthly income ($500–800: *β* = −0.079, *p* < 0.05; $800–1,000: *β* = −0.128, *p* < 0.001).

These findings highlight the interplay of clinical (oral and general health, HbA1c control), psychosocial (attitudes, education, family history), and structural (employment, income, healthcare-seeking behavior) factors in shaping OHRQoL in diabetic patients.

**Figure 5:**
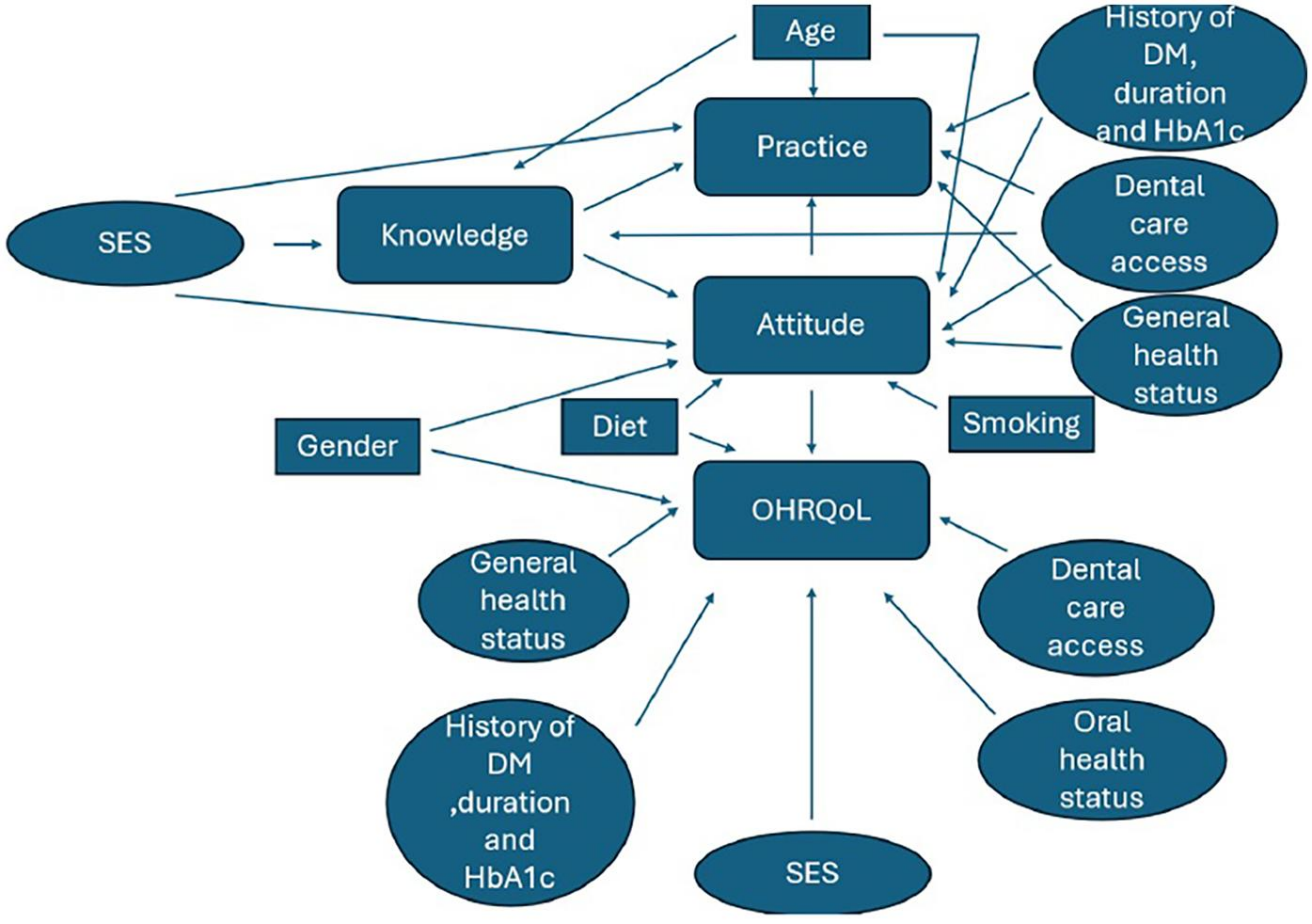
Modified model based on current study results. *SES (socio-economic status).

Figure 5 depicts the newly proposed conceptual model, which was constructed according to the significant findings and interrelationships among the study variables.

## Discussion

This study is the first to assess OHRQoL among diabetic patients in the West Bank and examine its relationship with knowledge, attitudes, practices, and sociodemographic and health-related factors. It employed a robust methodology, including a validated tool, standardized data collection, and a representative national sample, ensuring the findings are reliable and generalizable.

This study applied the Knowledge-Attitude-Practice (KAP) model to explore oral health and OHRQoL in diabetic patients. In line with previous studies (38–40), our findings show that even though diabetic patients demonstrated moderate knowledge and positive attitudes toward oral health, these did not consistently translate into proper oral hygiene practices. This suggests that knowledge and attitude alone are insufficient to change behavior. Interventions should therefore go beyond education to include strategies that facilitate behavior change, such as structured oral health programs, reminders, and improved access to dental care, to effectively promote better oral health practices among diabetic patients. A positive attitude was strongly linked to higher OHRQoL, over 60% of participants acknowledged the importance of oral health, this result is similar to other research on diabetic patients (25, 41).

Sociodemographic factors influenced outcomes: younger, educated, and higher-income participants had better oral health knowledge, as shown in previous literature about KAP in Tanzania and Pakistan (39, 42), while employed, educated females demonstrated more positive attitudes, consistent with results of previous study about KAP of diabetic patients in Saudi Arabia (43). However, knowledge did not always translate into good practices, likely due to competing diabetes management, lack of motivation, and limited access to dental care (39, 42). Better practices were linked to higher income and employment, similar to global findings (43), while older age was associated with poorer hygiene, possibly due to denture use, impaired motor skills, or cumulative oral health challenges (44, 45).

Controlled diabetes correlated with better attitudes and practices, reflecting the bidirectional relationship between diabetes and periodontal disease. Poor glycemic control exacerbates oral inflammation, periodontitis, and xerostomia, which in turn can worsen systemic glycemic control, as reported in other research for Genco et al. about infection and inflammation in periodontal and cardiovascular diseases (46). Despite this, only 43.5% discussed diabetes with a dentist and 32.2% with a physician, emphasizing the need for interprofessional collaboration. Local and global literature highlight that integrated care improves both oral health outcomes and glycemic control (47, 48), reducing risks of cardiovascular, kidney, and other complications (49).

Only 18% of participants attended diabetes education programs, highlighting a critical gap in public health outreach, consistent with previous study by Abbasi et al. in Malaysia (50). Access to dental care improved knowledge, attitudes, and practices (51), while preventive and aesthetic treatments motivated better hygiene (52). Positive lifestyles, including healthy diet and non-smoking, were linked to better outcomes, whereas current smokers had poorer attitudes, consistent with previous study in Tehran, Iran by Sadeghi et al. (53). These observations suggest that public health strategies targeting lifestyle modification, smoking cessation, and dietary counseling are essential for improving oral health behaviors in diabetic populations.

Poor OHRQoL in this study was mostly attributed to psychological discomfort, handicap, and social disability. Higher HbA1c levels, poor general and oral health, and low socioeconomic status were associated with worse disability domains (51, 52). Age, poor practices, and low knowledge further increased disability. These results highlight the need to address both biological and social determinants of health through health education, behavioral interventions, and socioeconomic support.

Participants who had not attended educational programs or communicated with providers had worse outcomes across OHIP−14 domains (54, 55). Interestingly, those who did attend reported higher psychological distress, possibly due to increased awareness of complications without adequate support, as also reported in prior studies (56, 57). Crisis-driven dental visits for pain or cosmetic reasons were linked to worse OHRQoL, reflecting reactive care-seeking patterns seen locally and globally (44).

Poor glycemic control (high HbA1c) correlated with worse OHRQoL across all domains (47), whereas higher education, income, positive attitudes, and better practices predicted improved outcomes (58, 59). Sociodemographic disadvantages—older age, unemployment, low income, and low education—were associated with poorer OHRQoL, consistent with both Palestinian and global studies (23, 49, 59). Self-reported oral issues and poor glycemic control worsened OHRQoL, while milder, non-medicated diabetes was linked to better outcomes and these results the same in UAE and Kuwait (60, 61). Interestingly, lack of family history predicted poorer OHRQoL—a novel finding. This may reflect reduced awareness, lower engagement in preventive care, and missed early interventions.

Overall, this study demonstrates that oral health in diabetic patients is influenced by clinical, behavioral, and socioeconomic factors. Integrating oral health into routine diabetes care, expanding education, and addressing social inequalities are critical, as supported by literature. Recommended public health interventions include structured diabetes and oral health education programs, improved access to preventive dental care and hygiene tools, lifestyle interventions such as healthy diet and smoking cessation, and policies promoting interprofessional collaboration between dentists and physicians. Implementing these strategies can enhance oral health practices, reduce complications, and improve OHRQoL among diabetic patients, particularly in low-resource settings like Palestine.

Importantly, these recommendations must be adapted to the realities of the Palestinian health system. Primary healthcare centers managed by the Ministry of Health (MoH) already serve as first-line providers for diabetes care and thus represent a practical entry point for integrating oral health screening and education. Low-cost interventions—such as training diabetes educators and nurses to deliver brief oral health messages, incorporating oral health checklists into diabetes visits, and engaging community health workers in rural areas—would be feasible within existing structures. Partnerships with local universities and dental schools could further support preventive outreach and patient education campaigns. Given financial and resource constraints, prioritizing preventive care, task-shifting, and interprofessional collaboration offer realistic pathways for improving oral health outcomes among Palestinian diabetic patients.

## Limitations and future research

This study's cross-sectional design limits causal inference, capturing associations at a single time point. Reliance on self-reported data may introduce recall or social desirability bias. As the study was conducted only in West Bank PHCs, findings may not generalize to Gaza or underserved populations outside PHC coverage. Future research should use longitudinal or mixed method designs and include diverse geographic and socioeconomic groups to better understand the determinants of oral health behaviors and OHRQoL among diabetic patients in Palestine.

## Conclusion

This study provides important insight into the OHRQoL of diabetic patients in the West Bank. While knowledge and positive attitudes were common, they did not consistently translate into optimal oral hygiene behaviors. Positive attitudes were associated with better OHRQoL, whereas poor outcomes correlated with limited healthcare access, low socioeconomic status, poor glycemic control, and psychological distress. Improving OHRQoL requires a holistic, patient-centered approach that integrates oral health into chronic disease management, promotes healthy lifestyles, and addresses structural and social barriers. Findings should be interpreted cautiously due to limited generalizability and modest explanatory power, suggesting that additional clinical, psychosocial, and structural factors likely influence OHRQoL.

## Data Availability

The original contributions presented in the study are included in the article/Supplementary Material, further inquiries can be directed to the corresponding authors.
