# Supplementary material for: Oral health KAP and their association with OHRQoL among type 2 diabetic patients in the West Bank, Palestine: a cross-sectional study
Source: Front Oral Health. 2025 Oct 31;6:1670923. doi: 10.3389/froh.2025.1670923 (PMC12642810; doi:10.3389/froh.2025.1670923)
Supplement: Supplementary file 1 [file Supplementaryfile1.pdf]

Oral Health KAP and Their association with Quality of Life Among Type 2 Diabetic Patients Aged ≥40 in the West Bank, Palestine: A Cross-Sectional Study

Iman Wahbeh [a], **Aesha L.E Enairat** [a] **Ihab Hemieid** [b] **Mahmoud Amro** [b] **Malak Abueed** [c] **Yazan Najem Hirzallah** [b] **Elham Kateeb** [d]

[a] Faculty of Graduate Studies, AL- Quds University, Jerusalem Palestine

[b] Faculty of Medicine, Al-Quds University, Jerusalem Palestine

[c] Faculty of Medicine and Health Sciences, An-Najah National University, Nablus, Palestine

[d] Oral Health Research and Promotion Unit, Al-Quds University, Jerusalem Palestine

Iman Wahbeh 1st and corresponding author

Elham Kateeb supervisor and co-corresponding author

***Correspondence:** iman.wahbeh@students.alquds.edu

ekateeb@staff.alquds.edu

Questions of the study tool were extracted from the literature(1–4) based on the proposed conceptual framework of the study's variables(5–7) (Fig 2). The validated Arabic version of OHIP-14 instrument was used, which has been previously translated and validated(8,9) (Table 9).

The questionnaire comprised five sections:

1. **Socio-demographic, Health status and Access to dental care**: This section collected data on participants' gender, age, marital status, family size, education level, income, residence, employment status, history of diabetes, chronic diseases, oral health issues, smoking habits, diet, and access to healthcare services (see Tables 1-5).
2. **Oral Health Knowledge variable**: A ten-question scale assessed participants' knowledge of the relationship between diabetes and oral health. Responses were categorized as correct or incorrect. The questions covered whether people with diabetes are more susceptible to gum diseases and supporting tissues, if there is a link between gum disease and increased blood sugar levels, and whether diabetes leads to bad breath. Additional topics included the susceptibility of diabetics to dry mouth and oral thrush, the incidence of tooth decay due to dry mouth, whether diabetes can cause tooth loss, and if regulating blood sugar can help prevent oral diseases. This section also assessed the knowledge about whether smoking is a risk factor for oral diseases in diabetics and if chronic gum infections are linked to cardiovascular diseases in diabetic patients (Table 6).
3. **Attitudes Towards Oral Health:** Six questions were used to evaluate participants' attitudes toward oral health using a 5-point Likert scale (Totally Agree, Agree,

Neither Agree nor Disagree, Disagree, Totally Disagree). The questions focused on the importance of oral care and dental visits, including whether taking care of oral health is as important as other parts of the body, the importance of brushing teeth in the morning and before bed, and the necessity of visiting the dentist at least once a year. This section also explored whether participants believe in consulting a dentist for oral conditions, the importance of healthcare teams providing information about diabetes complications related to oral health, and the need for diabetes doctors to refer patients to a dentist for regular examinations (Table 7).

4. **Oral Hygiene Practices**: This section covered six questions on self-care oral hygiene practices, including the frequency of toothbrushing, use of dental floss, fluorides, and mouthwash, as well as brushing techniques and duration. Participants' responses regarding toothbrushing frequency and flossing were categorized as "never," "often," "seldom," or "at least once daily." The use of fluoride and mouthwash was recorded as "yes," "no," "sometimes," or "I don't know." The technique of toothbrushing was assessed as either correct or incorrect (after the patient demonstrated the way of brushing in front of the interviewer) , and the duration of brushing was categorized as "less than one minute," "two minutes," "more than two minutes," or "not brushing"(Table 8).
5. **OHIP-14 Instrument**: The final section utilized an Arabic version of OHIP-14 instrument, which has been previously translated and validated(8,9) (Table 9).

OHIP-14 is the short form of OHIP-49 and widely used and accepted to measure the impact of OHRQoL. Arabic version of (OHIP-14) was used in this study(8,9), validated by previous articles, with a Cronbach's alpha coefficient of 0.80 for the Arabic version. In the literature, the α values for the seven subscales of OHIP-14 ranges from 0.75 to 0.81 (8). The format of the question was as follows, how often during the last 12 months have you had (impact item)

because of problems with your teeth, mouth, or dentures? The questionnaire included 14 questions related to the experience: articulation difficulties, degradation of taste, pain, discomfort during eating, self-consciousness, emotional tension, dissatisfaction with eating, interruption of eating, difficulty relaxing, embarrassment, irritability, inability to complete daily tasks, reduced satisfaction with life, and complete inability to work. The frequency of occurrence was assessed on a five-point Likert scale: 0 = never, 1 = seldom, 2 = sometimes, 3 = frequently, and 4 = very often. All values were summed to calculate a total OHIP-14 score, which can vary between 0 and 56, the higher the OHIP-14 score, the worse the OHRQoL. In the pilot study in this research project, the Cronbach's α coefficient for the OHIP-14 was 0.87.

Data Analysis

Descriptive and Normality Testing

Descriptive statistics, including means, standard deviations, frequencies, and percentages, were used to summarize participants' sociodemographic characteristics, diabetes history, general and oral health status, and access to healthcare services. The normality of the dependent variables—Knowledge, Attitude, Practice (KAP), and Oral Health-Related Quality of Life (OHRQoL)—was assessed using the Kolmogorov-Smirnov and Shapiro-Wilk tests. A p-value of less than 0.05 indicated a non-normal distribution, leading to the use of non-parametric tests for bivariate analysis. IBM SPSS Statistics version 26.0 was used for all statistical analyses.

Scoring and Composite Variable Construction

Knowledge

Knowledge scores were computed by awarding one point for each correct response and zero for incorrect answers. The total composite knowledge score ranged from 0 to 10, with higher scores indicating greater knowledge.

Attitude

Responses on a five-point Likert scale (totally agree, agree, neither agree nor disagree, disagree, totally disagree) were recorded as follows: "Totally agree" and "Agree" were coded as 1 (positive attitude). The remaining responses were coded as 0 (negative attitude).
The composite attitude score ranged from 0 to 6, with higher scores reflecting more positive attitudes.

Oral Hygiene Practices

Oral hygiene practices were categorized as follows:

- Toothbrushing and dental flossing: "At least once a day" was considered good practice (1 point), while less frequent practices were deemed poor (0 points).
- Mouthwash and fluoride use: "Yes" and "Sometimes" were classified as good practices (1 point), while all other responses were poor practices (0 points).
- Brushing technique: Correct technique was considered a good practice (1 point), while incorrect technique was considered poor (0 points).
- Brushing duration: A duration of two minutes was classified as good practice (1 point), while any other duration was classified as poor (0 points).

The total oral hygiene practice composite score ranged from 0 to 6, with higher scores indicating more favorable oral hygiene behaviors.

Dietary habits: Responses related to sugar was originally measured using a five-point Likert scale and subsequently recoded into a three-category scale reflecting dietary habit quality. In this recoding, higher scores indicated poorer dietary habits. Specifically, responses of "Never" and "Rarely" were assigned a score of 0, indicating very good habits; "Sometimes" was assigned a score of 1, indicating fair habits; and "Often" and "Always" were assigned a score of 2, indicating bad habits.

For the fruit and vegetable consumption question, which was phrased as 'Fruits and vegetables are not part of my diet habits' with response options of 'Never,' 'Rarely,' 'Sometimes,' 'Often,' and 'Always,' the recoding was adjusted so that higher scores reflected poorer dietary habits. In this case, 'Never' and 'Rarely' were considered indicators of good dietary habits and were assigned a score of 0. 'Sometimes' was assigned a score of 1, reflecting fair dietary habits, and 'Often' and 'Always' were considered indicators of poor dietary habits, with a score of 2.

Oral complications: "Yes" responses were assigned 1 point, while "No" and "Not sure" responses were assigned 0 points. The total composite score reflected the burden of oral health issues, with higher scores indicating more complications.

General health complications: A similar scoring approach was used, where "Yes" was assigned 1 point and "No" was assigned 0 points. Higher scores indicated a greater burden of systemic health complications.

Oral Health-Related Quality of Life (OHRQoL)

OHRQoL was assessed using the **Oral Health Impact Profile (OHIP-14)**, with total scores ranging from 0 to 56. Higher scores indicated poorer OHRQoL. The OHIP-14 consists of seven domains, each scored on a scale from 0 to 8, higher scores indicated poorer OHRQoL.

Bivariate Analysis

Bivariate analysis was conducted to examine relationships between key study variables:

- **Spearman correlation tests** were used to assess associations between OHIP-14 domains and predictor variables, including age, education, income, cumulative sugar intake, diabetes duration, and the composite variables of knowledge, attitude, hygiene practices, oral health complications related to diabetes, general health status, and dentalcare access (e.g., last dental visit).
- **Mann-Whitney U test** was used to compare differences between participants who had attended diabetes education programs and those who had not, as well as those who discussed their disease with physicians or dentists versus those who did not.
- **Kruskal-Wallis test** was conducted to assess differences in OHIP-14 domains based on the reason for the last dental visit.

Multivariate Analysis

A stepwise multiple linear regression was performed to examine the influence of study's predictor variables on OHRQoL (treated as a continuous dependent variable). Predictor variables included all significant variables from the **Mann-Whitney U**, **Kruskal-Wallis**, and **Spearman correlation** tests. Additional theoretically relevant variables based on prior literature and a conceptual model were included such as Knowledge score, Practice score,

Age, Smoking status, Family history of diabetes, Last dental visit, Attendance at diabetes educational programs. Since some variables were categorical, they were recorded into dummy variables, resulting in **51 predictor variables** included in the regression model. The stepwise regression approach was confirmed using **forward regression analysis**.

Collinearity and Model Fit

Collinearity diagnostics were conducted to ensure statistical validity: **Variance Inflation Factor (VIF)** values were all below 1.21 and **Tolerance values** were above 0.82, indicating no multicollinearity among predictors. The **goodness of fit** for the regression model was evaluated using the **adjusted R^2** value. A **significance level of $p < 0.05$** was applied for all statistical tests, with **two-tailed tests** used for hypothesis testing.

References

1. Alhajaji R. Oral Health Knowledge, Attitude and Practice among adults living with diabetes in Makkah City. J Res Med Dent Sci. 2022;10(1):495-+.
2. Ismaeil FM, Ali N. Diabetic Patients Knowledge, Attitude and Practice toward Oral Health. J Educ Pract [Internet]. 2013;4(20):19–25. Available from: http://www.iiste.org/Journals/index.php/JEP/article/viewFile/7895/7969
3. Mahzari MA, Oraibi OH, Shami AM, Shami MO, Thobab TY, Awlaqi AA, et al. Knowledge, Attitude, and Practice Regarding Diabetes Mellitus Among Type 2 Diabetic Patients Attending Primary Health Care Centers in the Jazan Region of Saudi Arabia. Cureus. 2022;14(9).
4. Mian RI, Rashidi FFH, Alshammary TM, Zubaidi S Al, Shammary F Al, Siddiqui AA, et al. Oral health-related knowledge and assessment of oral health status of diabetic patients attending dental clinics at college of dentistry, Hail, Saudi Arabia. J Contemp Dent Pract. 2020;21(1):78–82.
5. Zheng S, Zhao L, Ju N, Hua T, Zhang S, Liao S. Relationship between oral health-related knowledge, attitudes, practice, self-rated oral health and oral health-related quality of life among Chinese college students: a structural equation modeling approach. BMC Oral Health [Internet]. 2021;21(1):1–11. Available from: https://doi.org/10.1186/s12903-021-01419-0
6. Zhu W, Liang D, Petersen JD, Zhang W, Huang J, Dong Y. Relationship between diabetic knowledge, attitudes and practices among patients with diabetes in China: A structural equation model. BMJ Open. 2023;13(11).

7. Rav-Marathe KSR on the K-OF for DE and R, Wan TTH, Marathe S. a Systematic Review on the Kap-O Framework for Diabetes Education and Research. Med Res Arch. 2016;4(1):1–21.

8. Khalifa N, Allen PF, Abu-bakr NH, Abdel-rahman ME. Psychometric properties and performance of the Oral Health Impact Profile (OHIP-14s-ar) among Sudanese adults. 2013;55(2):123–32.

9. Osman SM, Khalifa N, Alhajj MN. Validation and comparison of the Arabic versions of GOHAI and OHIP-14 in patients with and without denture experience. 2018;1–10.
